# Ultrastretchable Elastic Shape Memory Fibers with Electrical Conductivity

**DOI:** 10.1002/advs.201901579

**Published:** 2019-08-28

**Authors:** Sungjune Park, Neil Baugh, Hardil K. Shah, Dishit P. Parekh, Ishan D. Joshipura, Michael D. Dickey

**Affiliations:** ^1^ Department of Polymer‐Nano Science and Technology BK21 Plus Haptic Polymer Composite Research Team Department of BIN Convergence Technology Chonbuk National University Jeonju 54896 South Korea; ^2^ Department of Chemical and Biomolecular Engineering North Carolina State University 911 Partners Way Raleigh NC 27695 USA

**Keywords:** elastic shape memory fibers, liquid metals, stretchable electronics

## Abstract

Herein, elastomeric fibers that have shape memory properties due to the presence of a gallium core that can undergo phase transition from solid to liquid in response to mild heating are described. The gallium is injected into the core of a hollow fiber formed by melt processing. This approach provides a straightforward method to create shape memory properties from any hollow elastic fiber. Solidifying the core changes the effective fiber modulus from 4 to 1253 MPa. This increase in stiffness can preserve the fiber in a deformed shape. The elastic energy stored in the polymer shell during deformation drives the fiber to relax back to its original geometry upon melting the solid gallium core, allowing for shape memory. Although waxes are used previously for this purpose, the use of gallium is compelling because of its metallic electrical and thermal conductivity. In addition, the use of a rigid metallic core provides perfect fixity of the shape memory fiber. Notably, the use of gallium—with a melting point above room temperature but below body temperature—allows the user to melt and deform local regions of the fiber by hand and thereby tune the effective modulus and shape of the fiber.

## Graphical abstract

**Herein, soft and ultrastretchable elastic shape memory fibers with electrical conductivity** are demonstrated fabricated by injecting liquid metal, gallium, into the elastic and hollow fibers. The ability to change the core of the fiber from liquid to solid allows an enormous modulus change and thus, excellent shape memory effects.

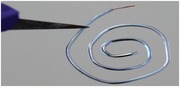

## 1 Introduction

This paper describes hollow elastomeric fibers with metallic cores that can be programmed into stable, temporary shapes at room temperature. The shape of the fibers recovers rapidly and elastically (i.e., with minimal viscous dissipation) when heated due to the ability of the cores to undergo a solid–liquid phase transition. We fabricated the conductive fibers by injecting a liquid metal, gallium, into stretchable hollow fibers formed by melt‐spinning a commercial thermoplastic elastomer. The ability to change the core of the fiber from solid (with a modulus of a metal) to a liquid (with a viscosity near water) allows for dramatic changes in mechanical properties as well as the ability to have shape memory effects. Although the mechanism differs, this effect is similar to shape memory polymers (SMPs),^1^, ^2^, ^3^, ^4^, ^5^, ^6^, ^7^, ^8^, ^9^, ^10^ which are fascinating materials that can be programmed to store and recover elastic energy in response to external stimuli. SMPs can be deformed at elevated temperature and then cooled to retain their temporary shape. Heating the polymer again to the elevated temperature allows the stored strain to relax back to the original, predeformed shape.

The use of metallic cores as a mechanism to retain elastic fibers in a temporary shape has several advantages relative to conventional shape memory polymers:

(1) Processability: Although there are a variety of molecular strategies to create shape memory polymers, not all shape memory polymers can be melt processed (e.g., due to temperature sensitivity, crosslinking, or poor melt flow properties). Here, any elastic polymer that can be melt processed into a hollow fiber can be rendered to have shape memory properties by simply injecting gallium into the core.
(2) Fixity: The ability of a strained SMP to hold its shape is characterized by “fixity” and its ability to return to its original shape is termed “recovery.” The use of a rigid (metallic) core provides nearly perfect fixity, as shown herein. By contrast, most conventional SMPs have some small amount of relaxation that lowers the fixity, although there are exceptions.^11^
(3) Electrical conductivity: The gallium core results in a fiber with metallic electrical conductivity.^12^ Conductive fibers have attracted considerable interest due to their ability to be integrated into clothing and fabrics.^13^, ^14^
(4) Triggering mechanisms: The use of a metal core allows new triggering mechanisms to release strain. For example, Joule heating or induction heating can heat the fibers above the melting point of gallium because of the electrical conductivity of the core.^15^ In addition, shape memory materials that can trigger at body temperature are relevant for biomedical applications.^16^ Plus, the fibers here trigger a shape memory response above a single temperature: the melting point of the metal. By contrast, shape memory polymers often respond over a relatively wide range of temperatures associated with the glass transition temperature.
(5) Drawing ability: It is possible to elongate, and thus, narrow the cross‐section of the fibers at low temperatures via postprocessing, such as melt processing or “drawing,” which is similar to the processes used to create optical fibers.^17^
(6) Mechanical properties: When solidified, the core gives the fibers metallic mechanical properties, which differ from those of polymers. The presence of the solid metal core can result in tough fibers via serial fracturing.^18^ The elastomeric shell can be deformed to very large strains (800%) when the core is in the liquid state. In addition, the ability to locally melt only portions of the core can tune the effective macroscopic mechanical properties (i.e., small macroscopic strains can create large local strains within the fiber).
(7) Recovery speed: Conventional SMPs often have relatively slow shape recovery due to their low thermal conductivity (heat is necessary to raise the temperature of the SMP) and viscous dissipation during relaxation. The response time of SMPs can be increased by using elastic polymers with crystalline domains that preserve a programmed state; when these domains melt, the stored elastic energy drives rapid shape recovery.^19^, ^20^ The fibers in this paper take this concept one step further by utilizing a core that can be converted to a liquid with a viscosity near that of water.^21^ The elastomeric shell, combined with a liquid core, minimizes viscous retardation during shape recovery. In addition, the high thermal conductivity of the metal helps speed up the heating step necessary to trigger the shape recovery.

The work here is inspired by the work of several groups, which we summarize briefly to clarify the novel aspects of the present work. Commercial rubber with stearic acid “wax” exhibits SMP behavior (notably 100% fixity and 95% recovery) in which the phase transition of the stearic acid triggers the SMP response.^11^ These materials have the appeal of being inexpensive and display faster recovery than conventional SMPs, but they are not electrically conductive and require a relatively high temperature for switching phases (75 °C). Low melting point alloy (LMPA)–elastomer composites—that is, porous elastomer foam filled with LMPA—store elastic energy upon deformation and release it upon melting the LMPA.^22^ These composites show modest changes in stiffness due to the discontinuous nature of the metal and do not have fiber form factors. Silicone fibers filled with an LMPA exhibit both rigidity‐tuning properties via Joule heating and the ability to preserve temporary shapes.^23^ The work here builds on this concept by quantitatively characterizing the SMP behavior. In addition, the work here takes advantage of the fact body temperature is sufficient to melt the gallium core due to the low melting point of the metal (29.8 °C), thus enabling localized melting (and deformation) of the core using heat from fingers (for example).

Soft and stretchable conductive fibers with variable stiffness and shape memory may find applications in stretchable electronics,^24^ soft robotics,^25^, ^26^ and wearable devices.^27^, ^28^ The fiber form‐factor can create complex geometries via twisting and elongating and can undergo large deformation while retaining electrical continuity.^29^ Here, we characterize these fibers as shape memory materials and demonstrate their interesting properties.

## 2 Results and Discussion

**Figure 1:**
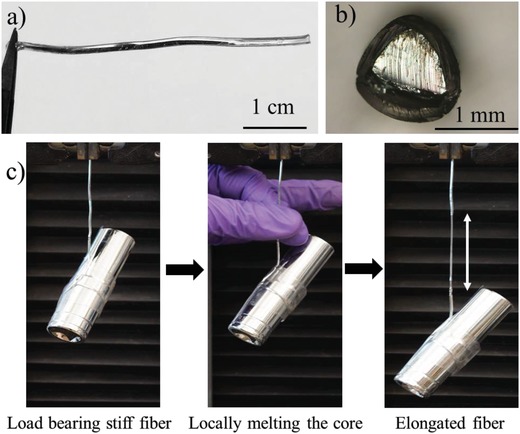
a) Photograph of a hollow elastic fiber filled with solid gallium, and b) a cross‐sectional view showing a gallium core in the fiber. c) A stiff fiber (left) can support a 200 g mass. Locally melting the core using body heat (middle) allows the fiber to locally deform (right) under the load. Removing the source of heat (here, fingers) allows the core to resolidify at room temperature in the extended state.

We injected liquid gallium into a hollow elastomeric fiber composed of a triblock copolymer, poly[styrene‐*b*‐(ethylene‐*co*‐butylene)‐*b*‐styrene] (SEBS).^29^ Despite the high tension of liquid metal, it stays within the fiber due to the presence of a thin oxide skin (≈3 nm) on its surface.^30^ Gallium is a supercooled liquid metal at room temperature;^31^ that is, it stays in the liquid state below its freezing point (29.8 °C). Supercooled liquids typically require a seed crystal or nucleus to form a solid. To provide a nucleation site in the liquid gallium, we inserted a gallium‐coated copper wire with the tip prewetted with hydrochloric acid. Below 29.8 °C, the metal freezes, starting at the nucleation site and propagating along the fiber. It was readily apparent which portion of the gallium was frozen by simply touching the outside of the fiber. Thus, it was possible to estimate the volumetric propagation of the solidification along the length of the fiber as (0.1–2.3) × 10^−4^ cm^3^ s^−1^ depending on the temperature conditions. The resulting solid core holds the fiber in stable, temporary shapes (**Figure** **1**a,b). The fiber is load bearing (Figure 1c) when the core is in the stiff state. When heated above the melting point, which can be accomplished with body heat, the fiber softens and deforms under the load.

**Figure 2:**
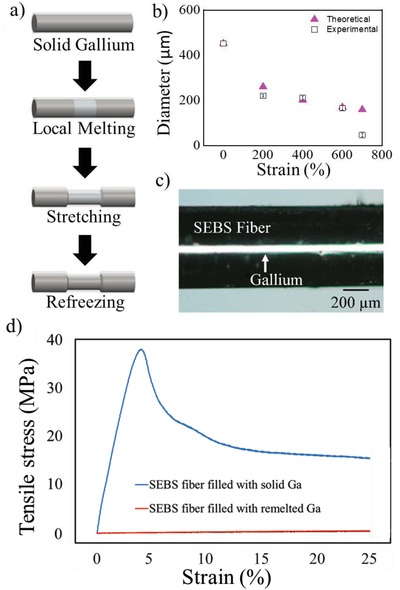
a) Schematic illustration showing the preparation of thin cross‐sectional metallic wires via local phase manipulation of liquid metal and subsequent stretching of the fiber, b) plot of the average cross‐sectional diameter of solidified gallium wires as a function of the strain applied to the fiber over five measurements, c) optical microscope image of the thinnest cross‐sectional metallic wire, and d) tensile stress–strain plots of the polymeric fibers with variable stiffness depending on phase of the metallic core. Tensile stress–strain plots of an empty SEBS fiber and liquid Ga filled fiber are nearly identical, as shown in Figure S1 of the Supporting Information.

We used this process of locally melting the core to create thin cross‐sectional metallic wires of a controllable diameter. As shown in Figures 1c and 2a, the solidified gallium in the fiber locally melts at body temperature to transform its phase from solid to liquid due to its relatively low melting point. The polymeric fiber with a liquid metal core locally elongates during stretching. Upon returning to room temperature, the liquid gallium refreezes due to the solid gallium at each end of the fiber acting as a seed point.^32^ However, if the gallium melts completely, it remains supercooled as a liquid at room temperature. Inserting a small copper wire or other nucleation source into the gallium at the tip of the fiber allows the gallium to resolidify at room temperature.

To quantify how this local elongation process affects the geometry of the wire, we solidified a Ga core with a diameter of 481 µm and length of 7.50 cm between two grips in an Instron extensometer. We melted a 2.5 cm long region and then stretched the fibers at 200 mm min^‐1^ to a predetermined strain. After the wires resolidified, we measured the diameter of the locally elongated region, and plotted it versus the strain of the locally molten region (which assumes deformation is localized entirely to that region). Figure 2b plots the diameters of the gallium wires as a function of strain. The measured values generally agree with the theoretical values calculated by assuming a conservation of volume of the metal. Each data point is an average of five repeat experiments, with the standard deviations represented by the bars. The outlier at 700% strain is likely due to the collapse of the cross‐section of the elastomeric shell based on the fact we previously observed a spike in resistance of these same fibers at this strain.^29^ The elongation procedure eventually leads to more than a 90% reduction of the diameter of the metallic wire, as it decreased from 481 to 47 µm. As shown in Figure 2c, metal still appears to uniformly fill the stretched polymeric fiber without any disconnections due to the intrinsic soft and fluidic nature of the liquid metal. This allows for a convenient and simple method of lengthening and narrowing wires near room temperature.

The mechanical properties of the fibers can be tuned by manipulating the phase of local regions of the fibers. While fully in the solid state, the fibers exhibit stress–strain profiles as seen in Figure 2d and as previously reported in the literature.^18^, ^23^ However, melting a portion of the solid core, as depicted in **Figure** **2**a, localizes the strain. This gives the ability to tune the effective mechanical properties (i.e., stress vs strain behavior) of the fibers based off of the length of the locally melted metal.

To quantify the mechanical properties of the fibers, we performed tensile stress‐strain measurements with both solid and liquid cores. Although the polymeric fiber can elongate up to 800% before breaking, we only show data to 25% strain to highlight the differences in the elastic moduli of the fibers. As shown in Figure 2d, the solidified liquid gallium causes the otherwise elastomeric fiber to become stiff. The rigid core plastically deforms (and ultimately fractures near 125% strain).^18^ The fibers filled with liquid gallium exhibit a similar mechanical response as the hollow polymeric fiber (Figure S1, Supporting Information).^29^ **Table** **1** reports the moduli of the fibers in the two states, which vary from 4.5 to 1253.6 MPa from liquid to solid, respectively.

**Table 1:** Comparison of mechanical properties of thermally activated stiffness‐tuning materials

Materials	Range of elastic modulus [MPa]	Difference [MPa]	Stiffness ratio
Gallium‐filled elastomeric fiber	4.5–1253.6	1249.1	278
LMPA‐filled silicone fiber^23^	1.2–887.8	886.6	740
LMPA‐filled PDMS microchannel^33^	1.5–40.0	38.5	27
Carbon black‐filled cTPE^34^	1.5–37.0	35.5	25
EGaIn‐coated cTPE^35^	0.7–10.4	9.7	15
LMPA‐imbibed silicone foam^22^	0.1–1.8	1.7	18

Previously, various rigidity‐tuning materials have been developed, including SMPs^11^, ^36^, ^37^ and LMPAs.^22^, ^23^, ^35^, ^38^ As compared in Table 1, the SEBS fiber filled with gallium reported here shows a large change of modulus (4.5 to 1253.6 MPa). The most relevant comparison of this work is to LMPA‐filled silicone fibers, which achieved a change from 1.3 to 887.8 MPa via solidification of a metal core. That prior work used silicone (which is approximately three times softer than the SEBS used here) and a lower ratio of metal to polymer (0.54 mm inner diameter and 1.03 mm outer diameter vs 0.91 inner and 1.2 mm outer diameter in this work). The consequence of these differences are summarized in Table 1.^23^

**Figure 3:**
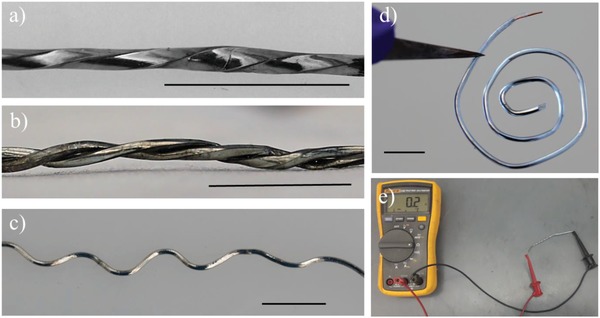
Conductive fibers with various geometries created by melting the metallic core, shaping the fiber, and allowing the metal to freeze. a) Twisted fiber, b) a pair of fibers intertwined in a double helix c) corrugated fiber, d) spiral fiber, and e) a voltmeter showing the fibers have metallic electrical conductivity. The scale bar is 1 cm.

We used the process of locally melting the core using body temperature and spontaneously resolidifying the metal to create various geometries of metallic wires. **Figure** **3** demonstrates the manipulation of polymeric conductive fibers with a metallic core into complex geometries such as twisted, spiral, helical, and corrugated structures by local melting the core and subsequent shaping of the fiber. This method is also capable of fabricating freestanding complex metallic wires by dissolving away the encapsulated polymer without involving either high temperature sintering or additional equipment.

**Figure 4:**
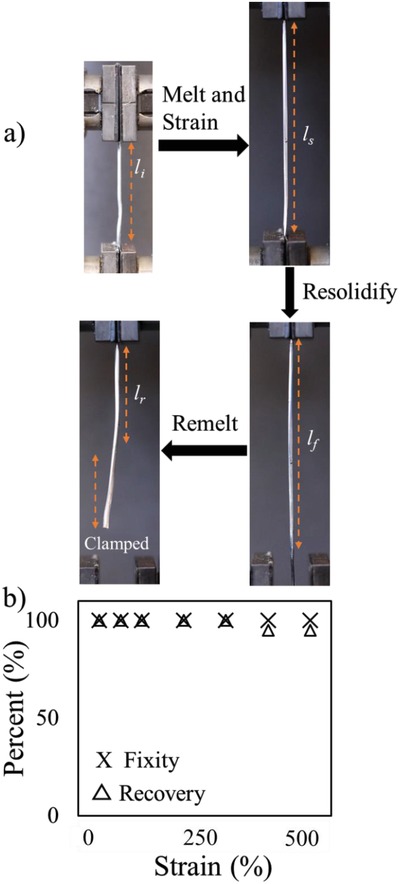
a) Experimental procedure for characterizing the shape memory behavior of the elastomeric stretchable fibers with a metallic core. The initial length between the grips is *l*_i_. After melting the core, the fiber is then elongated to length *l*_s_. At room temperature, the core freezes and holds the fiber at a length *l*_f_ upon releasing the bottom grip. Upon mild heating, the core melts and the fiber returns to a length *l*_r_. Note that after remelting the core, the image shows both the portion of the fiber that was once between the grips and the clamped portion that was once within the grip. b) Shape memory behavior as a function of strain.

Deformed fibers store elastic energy that can cause the fiber to relax back to its original shape when the core melts. This ability allows the fibers to have shape memory behavior. We systematically characterized the shape memory behavior of the SEBS fiber filled with gallium in terms of shape‐fixity and recovery under uniaxial tension using an Instron. This simple experimental setup allows rapid stretching with accurate strain control. As shown in **Figure** **4**a, an Instron clamped both ends of the SEBS fiber filled with the solidified gallium. We melted the unclamped area using body heat and subsequently stretched. Once elongtaed, the liquid gallium resolidified. After complete resolidification, we kept the stiff fiber at room temperature for one day and then characterized the shape‐fixity capability (*F*) by using Equation (1). Once the shape‐recovery process was activated by the phase change of the metallic core from solid to liquid upon heating, we characterized the shape‐recovery capability (*R*) using Equation (2), 11, 39

(1) $$F\;=\;\frac{\varepsilon_{f}}{\varepsilon_{s}}\;\times \;100$$

(2) $$R\;=\;\frac{\varepsilon_{f}\;-\;\varepsilon_{r}}{\varepsilon_{f}\;-\;\varepsilon_{i}}\;\times \;100$$

where strain is defined as ε_a_ = (*l*_a_ *– l*_i_)/*l_i_* and *l*_i_, *l*_s_, *l*_f_, *and l*_r_ correspond to the lengths of the initial fiber, stretched fiber, fixed fiber, and recovered fiber, respectively (note: subscript “a” represents s, f, or r) .

Figure 4b shows fixity and recovery as a function of strain. These conductive polymeric fibers show perfect shape‐fixity capability, i.e., 100% fixity. These results indicate that the use of a metallic core allows the fiber to retain a temporary geometry while storing elastic energy. The fibers also exhibit excellent (full) shape recovery performance by melting the gallium core in the fiber. They exhibit perfectly elastic shape memory characteristics of 100% recovery up to 300% strain, and 95% recovery at strains higher than 400% presumably due to plastic deformation of the elastomer.^29^

The fiber can recover its shape rapidly, as shown in Video S1 of the Supporting Information. When a fiber “frozen” (initially at room temperature) in a spiral geometry soaks in water at moderate temperature (32 °C), it retains the shape for 15 s while the gallium melts. After 15 s the fiber quickly deforms to recover its original shape. This shape‐recovery actuation is dramatically enhanced when we soaked the fiber in water at 65 °C; the fiber deforms as soon as it touches the warm water (Video S2, Supporting Information). **Table** **2** compares the speed of recovery to those of conventional shape memory materials. The elastic shell of the fiber causes the rapid activation of the shape memory with minimal viscous dissipation. Therefore, the recovery time is limited only by how long it takes to heat the fiber above the melting point of the metallic core. As shown in Table 2, the metallic core exhibits a higher thermal conductivity (40.6 W m^−1^ K^−1^) as compared to those of conventional shape memory materials (0.15–0.3 W m^−1^ K^−1^).^4^ Note that Table 2 reports the thermal conductivities and melting temperatures of the active components that enable the recovery (for this work, the Ga core).

**Table 2:** Comparison of phase‐changing temperature and mechanical properties of thermally activated stiffness‐tuning materials

Materials	Activation time [s]	Switching temperature [°C]	Shape memory behavior		Thermal conductivity [W m^−1^ K^−1^]
			Fixity	Recovery	
Gallium‐filled elastomeric fiber (present work)	2–16	>30	100%	95–100%	40.6
EGaIn coated cTPE^35^	2–40	>75	Not reported		26.4
LMPA‐filled silicone fiber^23^	29	>62	Not reported		18.0
LMPA‐imbibed silicone foam^22^	Not reported	>62	Not reported		
LMPA‐filled PDMS channel^33^	1	>47	Not reported		
Natural rubber with stearic acid^11^	15	>75	100%	95%	Not reported
Carbon black‐filled cTPE^34^	6	>75	Not reported		2.0

Because of the stabilizing effect of the oxide layer, it is possible to partially fill a hollow fiber with a plug of metal that is shorter in length than the rest of the fiber. Melting or solidifying this plug can also tune the effective macroscopic mechanical response of the fiber. Figure S2 of the Supporting Information demonstrates the ability to change the effective modulus of the fibers by a phase transition of a metallic plug. As shown in Figure S2a,c (Supporting Information), both the liquid metal core and the polymeric shell elongate during stretching of the fiber. However, the solid metal plug and the surrounding elastomer shell do not elongate significantly relative to the hollow portions of the fiber (Figure S2b,d, Supporting Information).^18^ Thus, when the metal plug is solid, the empty regions of the fiber experience a higher local strain (relative to the global strain) and therefore the fiber has a higher apparent (effective) modulus. Unlike the shape memory demonstration (cf. Figure 4), fibers containing a plug of solid metal require application of stress to keep the empty portions of the fiber elongated, which is a key distinction. Held in this elongated (stressed) state, melting the solid metal plug allows for the entire elastomeric shell to strain uniformly, thereby reducing the stress on the fiber, as demonstrated in Figures S3 and S4 (Supporting Information). This experiment was performed with silicone fibers, thereby demonstrating the versatility of the concept beyond thermoplastic elastomers. Lowering stress via phase manipulation, i.e., melting, of a solid metal core can be applied to various metal filled polymer fibers.

## 3 Conclusions

This paper describes soft and stretchable, elastic shape memory fibers with electrical conductivity fabricated by injecting liquid gallium into elastic and hollow fibers. The ability to change the core of the fiber from liquid to solid at body or near room temperature allows an enormous modulus change in the range of 4.5 to 1253.6 MPa and the ability to have shape memory effects. The use of a rigid metallic core provides perfect fixity, i.e., the ability to retain a deformed temporary shape. Elastic energy stored in the fiber causes the fiber to relax back to its original straight shape rapidly upon melting the gallium core. The thermal conductivity of the metal and the elastomeric nature of the shell minimize the time needed for shape recovery. These shape memory fibers have remarkable properties including metallic electrical and thermal conductivity, enormous change of stiffness, and excellent shape memory ability. Thus, they may be suitable for new applications in soft robotics and prosthetics. In addition, this approach to shape memory should extend beyond the uniaxial fibers (described here) by controlling the phase of metal in 2D or 3D vasculature inside elastomeric structures.

## 4 Experimental Section

*Fabrication of Elastomeric Shape Memory Fiber with Electrical Conductivity*: The elastomeric fiber used for this research was primarily a triblock copolymer, SEBS, hollow fiber having an outer diameter of 1.20 mm made by melt spinning a commercial thermoplastic elastomer (Kraton G1643). SEBS fibers with two different diameters were used. The fibers with an inner diameter of 481 µm were used in characterizing shape memory behavior and creating the thinnest metallic wires while the fibers with an inner diameter of 917 µm were used for measuring mechanical properties. Silicone fibers of inner diameter 1.10 mm and an outer diameter of 1.20 mm were used to demonstrate the ability to reduce stress in fibers and to demonstrate the versatility of the method beyond a single family of elastomers. To fabricate the conductive fiber, liquid metal (gallium) was injected into the hollow fiber using a syringe at room temperature. To solidify the liquid metal core in the fiber, a gallium‐coated copper wire was inserted into the core. The copper wire acts as a nucleation point for the gallium to crystallize, leading to a solid core. To create thin cross‐sectional wires, a small portion (2.5 cm) in the middle of the stiff fiber was melted using heat from contact with a finger for 2 min. Once the metallic core melts, the fiber was deformed to create various geometries. Complex geometries, such as a spiral and corrugation, were created by deforming the fiber into physical molds prior to freezing, which could occur at room temperature or accelerate by placing samples in a freezer.

*Characterization*: To characterize mechanical properties, an Instron 5493 with a 1 kN load cell was used. The stress–strain were repeated five times to deduce a mean value and a standard deviation for all reported modulus values. Two hydraulic grips held 1 in. sections of the fiber and stretched at a constant rate of 3 mm min^−1^ until it reached its failure. Cross‐sectional views of the fibers filled with metallic core were characterized and examined by an Olympus BX51 Microscope.

## Conflict of Interest

The authors declare no conflict of interest.

## Supporting information

Supplementary

Supplementary

Supplementary
