# Supplementary material for: Ultrastretchable Elastic Shape Memory Fibers with Electrical Conductivity
Source: Adv Sci (Weinh). 2019 Aug 28;6(21):1901579. doi: 10.1002/advs.201901579 (PMC6839750; doi:10.1002/advs.201901579)
Supplement: Supplementary file 1 — Supplementary [file ADVS-6-1901579-s002.pdf]

Copyright WILEY-VCH Verlag GmbH & Co. KGaA, 69469 Weinheim, Germany, 2019.

ADVANCED SCIENCE
Open Access

Supporting Information

for *Adv. Sci.,* DOI: 10.1002/advs.201901579

Ultrastretchable Elastic Shape Memory Fibers with Electrical Conductivity

*Sungjune Park, Neil Baugh, Hardil K. Shah, Dishit P. Parekh, Ishan D. Joshipura, and Michael D. Dickey**

Copyright WILEY-VCH Verlag GmbH & Co. KGaA, 69469 Weinheim, Germany, 2019.

Supporting Information

Ultrastretchable Elastic Shape Memory Fibers with Electrical Conductivity

*Sungjune Park, Neil Baugh, Hardil Shah, Dishit P. Parekh, Ishan Joshipura, and Michael D. Dickey**

Mechanical characterization of the fibers: To characterize mechanical properties of the fibers, we used an Instron 5493 with a 1 kN load cell. Two hydraulic grips held 1-inch long fibers and strained them at a constant rate of 3 mm/min until they failed. The hollow polymeric fibers exhibit a similar mechanical response as the fibers filled with liquid gallium (Figure S1 and 2d). Additional comparisons may be found in our previous study on liquid-filled elastomeric fibers, which shows that the liquid core has minimal impact on the mechanical behavior of the fibers relative to empty fibers.[1]

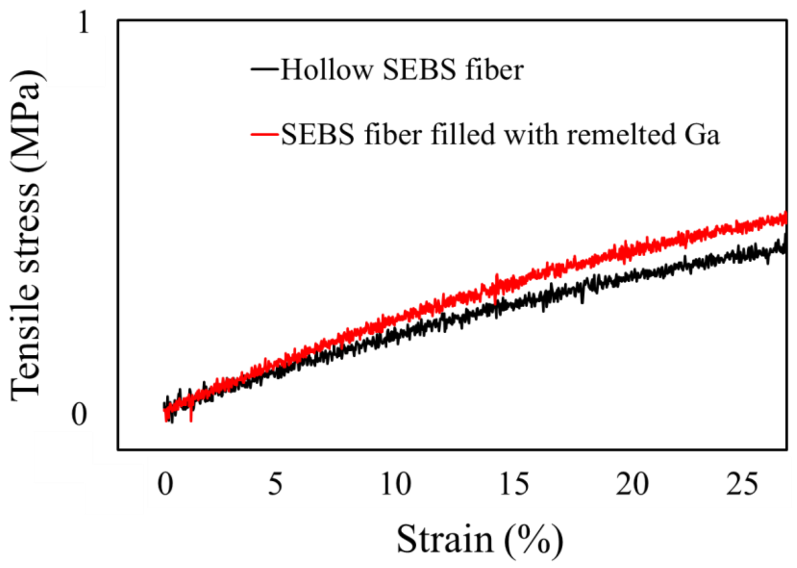

Figure S1. Tensile stress-strain plots of the hollow polymeric fiber show nearly identical behavior relative to liquid-filled fibers.

Demonstration of locally tunable modulus of the fiber: To demonstrate the ability to locally change the modulus of the elastomeric fibers by a phase transition of the metallic core, the SEBS fibers were partially filled with a plug of liquid metal (length L_0) in the middle of the fiber. The fiber was stretched with the metal in either the liquid or solid state. Both the liquid metal plug and the polymeric fiber simultaneously elongate during stretching due to the fluidic nature of the liquid metal and the elastic nature of the polymeric shell (Figure S2a and c). However, in the solid state, the metal does not significantly elongate, yet the empty regions of the fibers stretch readily (Figure S2b and d).

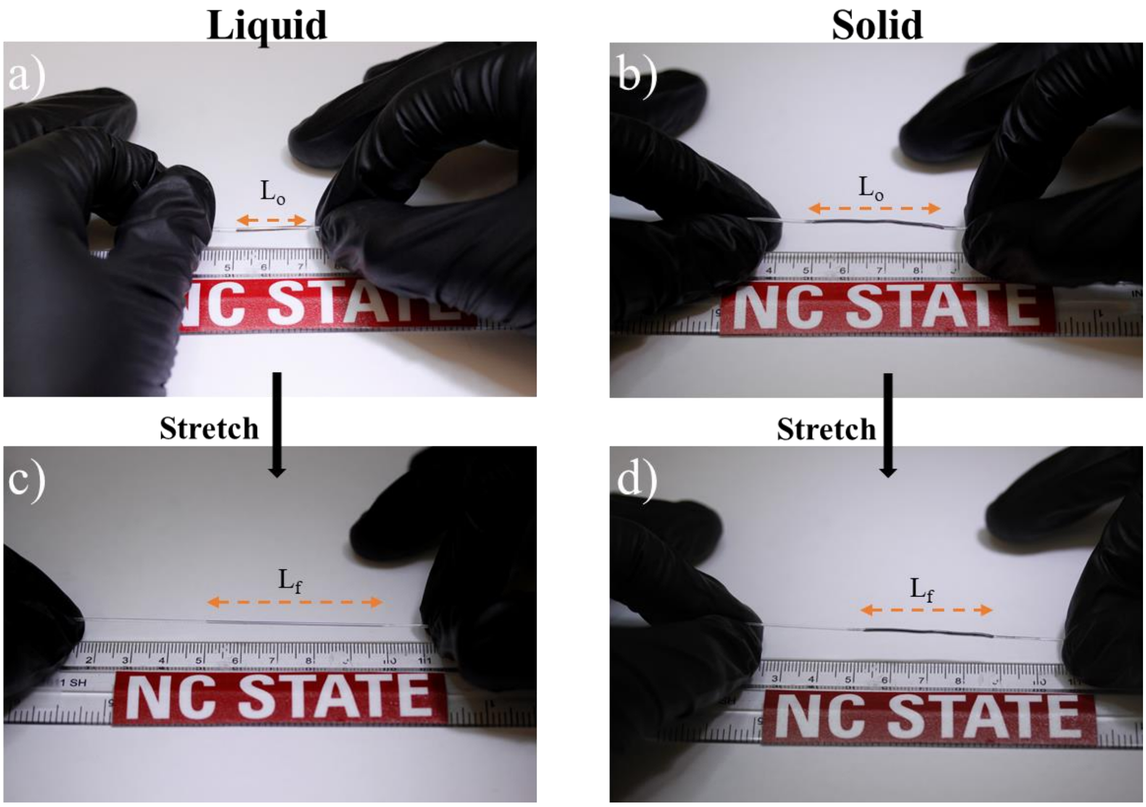

Figure S2. Photos of two different fibers. One with a liquid plug of metal in the core (left column), and one with a solid plug (right column). (a,c) SEBS fiber partially filled with a plug of liquid gallium in the middle of the fiber (a) before (L_0 = 2 cm) and (c) after stretching (L_f = 5.9 cm). (b,d) SEBS fiber partially filled with a plug of solid gallium in the middle of the fiber (b) before (L_0 = 3.7 cm) and (d) after stretching (L_f = 3.7 cm). In the solidified sample, most of the elongation occurs on the two empty end regions, which effectively increases the stress needed to achieve a desired global strain (i.e. the effective modulus increases).

Characterization of the decrease in stress for a fiber at constant strain: We partially filled silicone fibers with a plug of liquid metal and then elongated the fibers with the metal in the solid or liquid state. The samples were strained at 3 mm/min to 18% strain using an Instron 5493 with a 1 kN load cell. The samples were held at this strain for one minute and then relaxed to 0% strain at a rate of -3 mm/min. The fibers were then strained again at 3 mm/min to 18% strain and held for one minute while heat was applied from a hot air gun (Figure S3). After cooling for one minute, the liquid-filled fibers were then relaxed at -3mm/min to 0% strain. Figure S4 plots the second strain cycle to eliminate artifacts that may occur due to hysteresis in the first cycle. The length of the fiber between the grips, including the metal filled regions served as the gauge length for the calculation of strain. We speculate that the similarities in the stress experienced at low strains by the fibers filled with solid metal and fibers filled with liquid metal is due to slipping of the encasing polymer around the solid metal plug, which we have observed previously.[2]

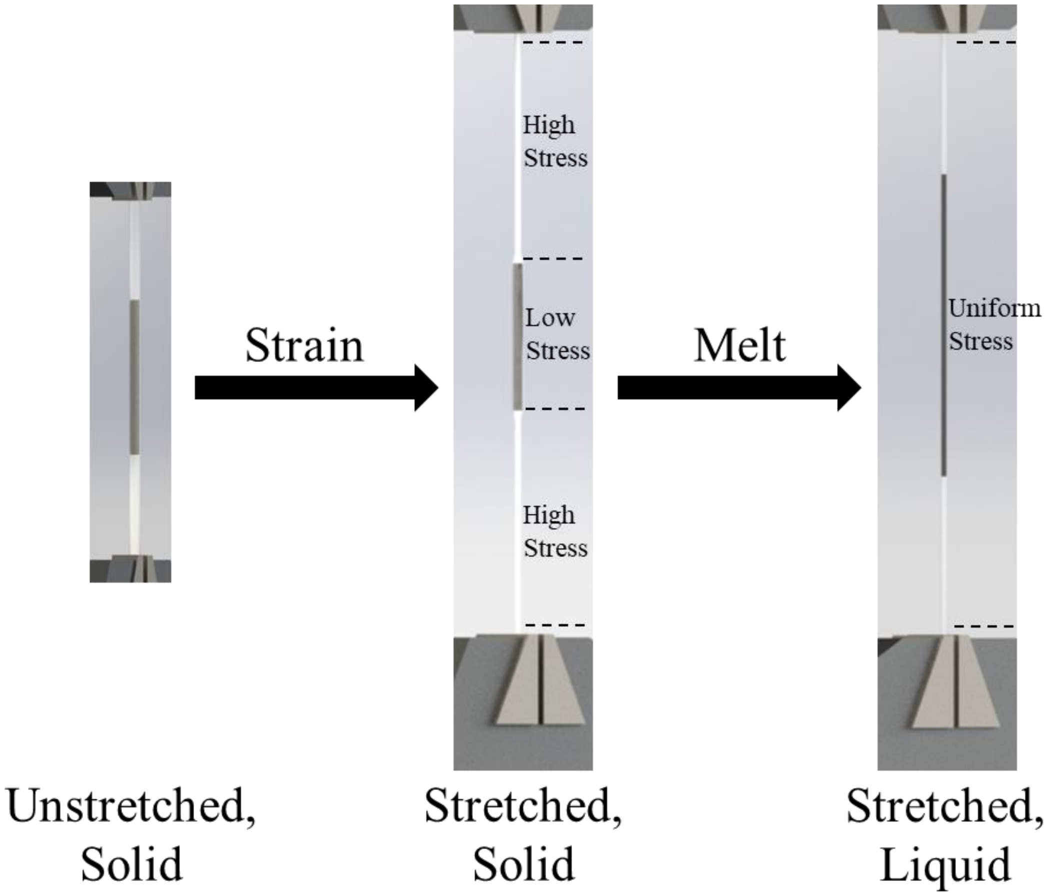

Figure S3. Conceptual images showing the effects of a plug of metal in the fiber. When the fiber is elongated between two grips, the portion of the fiber with the solid metal does not change shape significantly, and thus, strain is enhanced in the empty regions of the fiber relative to the overall strain. This strain results in high stress in the empty portion of the fiber relative to the stress experienced by the polymer shell surrounding the metal plug. Upon heating (melting) of the metal (which maintaining macroscopic strain), the strain within the fiber redistributes so that the local strain experienced by the polymer throughout the fiber is identical to the overall strain, and thus, the overall stress decreases.

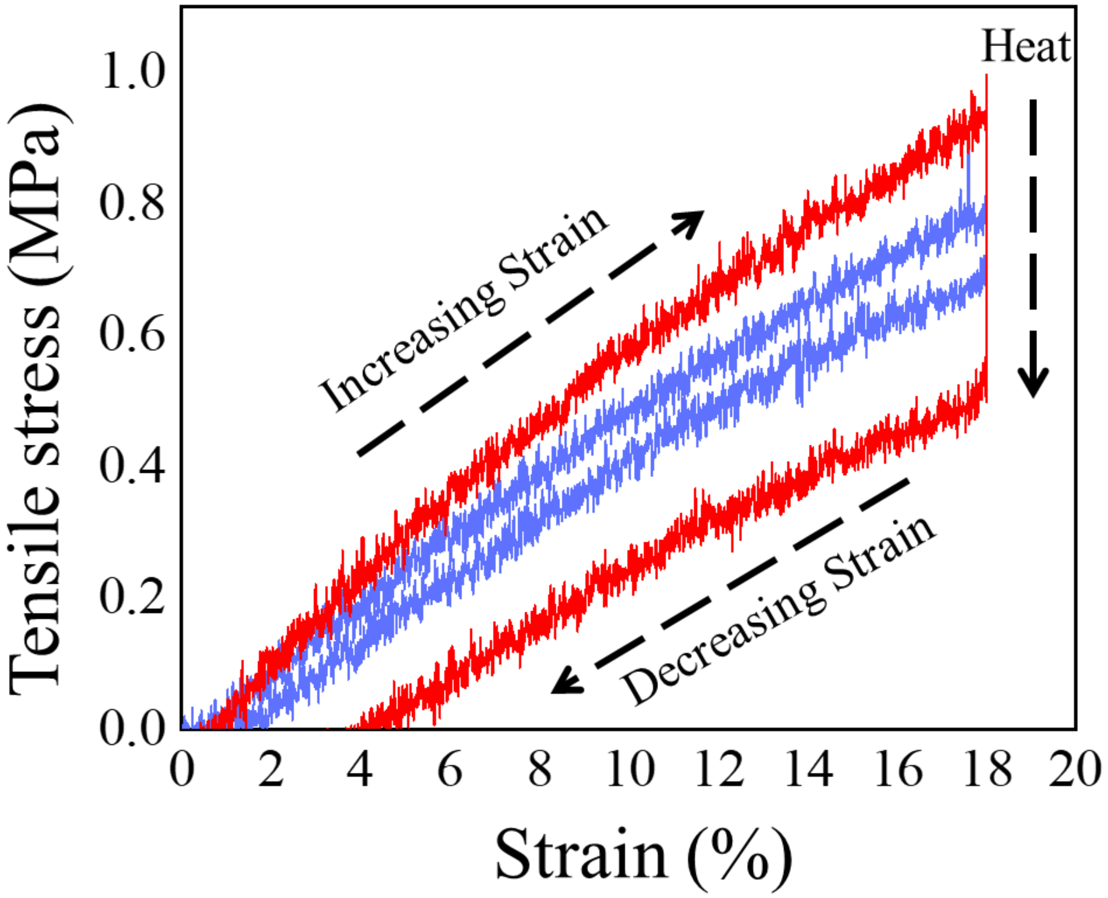

Figure S4. Tensile stress versus strain plot for a silicone fiber filled with a plug of metal. The red data shows a fiber that had a solid plug of metal before increasing the strain. When held at a constant strain (18% here) and heated, the stress decreases due to melting of the core. The blue data shows a fiber in which the plug started out as liquid and remained liquid throughout. The heating step causes the stress to decrease for reasons we do not fully understand, but may have to do with polymer relaxation during the heating. The heating step consisted of using a heat gun on the fiber for 1 minute. In both cases, the stress decreased upon heating, but the amount of decrease was greater for the fiber with a solid core.

Video S1. Shape recovery actuation of a fiber near room temperature: This video shows shape recovery of a fiber. The stiff fiber (with a frozen gallium core) in a spiral geometry is soaked in water at 32 °C. The fiber recovers its original straight shape after the gallium melts over the course of 15 s.

Video S2. Shape recovery actuation of a fiber at elevated temperature: This video shows rapid shape recovery of a fiber by heating well-beyond room temperature. The stiff fiber (with a frozen solid gallium core) is soaked in water at 65 °C. The fiber deforms as soon as it touches the water. We attribute this fast actuation to the rapid melting of gallium in warm water and the elastic shell, which has minimal viscous dissipation during relaxation.

References

[1] S. Zhu, J.-H. So, R. Mays, S. Desai, W. R. Barnes, B. Pourdeyhimi, M. D. Dickey, *Adv. Funct. Mater.* **2013**, *23*, 2308.
[2] C. B. Cooper, I. D. Joshipura, D. P. Parekh, J. Norkett, R. Mailen, V. M. Miller, J. Genzer, M. D. Dickey, *Sci. Adv.* **2019**, *5*, eaat4600.
